# Does formal public transport serve the city well? The importance of semiformal transport for the accessibility in Medellín, Colombia

**DOI:** 10.1371/journal.pone.0321691

**Published:** 2025-04-21

**Authors:** Dorothee Stiller, Michael Wurm, Marta Sapena, Simon Nieland, Stefan Dech, Hannes Taubenböck

**Affiliations:** 1 Earth Observation Center (EOC), German Aerospace Center (DLR), Oberpfaffenhofen, Germany; 2 Image Processing Laboratory, Universitat de València, Valencia, Spain; 3 Institute of Transport Research, German Aerospace Center (DLR), Berlin, Germany; 4 Department of Remote Sensing, Institute for Geography and Geology, Julius-Maximilians-Universität Würzburg, Würzburg, Germany; 5 Department of Global Urbanization and Remote Sensing, Institute for Geography and Geology, Julius-Maximilians-Universität Würzburg, Würzburg, Germany; Chang'an University, CHINA

## Abstract

Accessibility to public transport is a fundamental component of connecting individuals to urban services. Guided by the UN Habitat Sustainable Development Goal 11.2, which aims to ensure accessible, safe, affordable, and sustainable transport systems for all, our study focuses specifically on accessibility as a key dimension of achieving this goal and its implications for social and spatial equity. In this study, we employ the walking distance indicator proposed by the responsible working group of UN Habitat to calculate accessibility to public transport. Because underlying population data are an essential parameter for the indicator, we compare three distinct population datasets – cadaster-based population data, remote sensing-based population data, and a global dataset – to investigate spatial variations in accessibility across the city of Medellín, Colombia. Furthermore, we examine the impact of both formal public transport and the local semiformal minibus system (paratransit), analyzing differences across formal and informal settlement types of the city, as well as the influence of socio-economic factors. Our findings suggest that remote sensing based population data can serve as a valuable data source, albeit with limitations for global population data. Particularly, our results highlight the significance of the semiformal local minibus system in enhancing accessibility to public transport, despite ongoing expansions of the metro system by responsible authorities, which have led to considerable improvements in accessibility. Notably, we observe that residents with lower socio-economic status and those living in informal settlements experience longer walking distances to public transport stops, highlighting spatial and socio-economic disparities in accessibility. Overall, our study underscores the complex interplay between transport infrastructure, socio-economic factors, and urban development, highlighting the need for targeted interventions to address spatial and socio-economic disparities in public transport accessibility.

## 1. Introduction

Accessibility to public transport is a crucial facet of contemporary urban mobility. Ensuring accessibility to public transport not only promotes social inclusion but also fosters equal opportunities across various segments of society [1]. Moreover, it is crucial in connecting people to employment, education, healthcare, and other essential services [2]. Accessibility in general is conceptualized as the degree of convenience with which individuals can access specific locations of opportunities through the utilization of one or more modes of transport [2–4].

In 2015, the United Nations (UN) initiated the 2030 Agenda for Sustainable Development along with 17 Sustainable Development Goals (SDGs). The overarching aim was to advance global economic, social, and ecological sustainability [5]. Within the SDG 11 ‘Sustainable Cities and Communities’ seven targets are formulated including target 11.2 which focuses on enabling ‘access to safe, affordable, accessible and sustainable transport systems for all’ until 2030 [5]. To measure the status and progress towards target 11.2 on a global level, UN Habitat uses a distance-based indicator, which estimates the proportion of the population with convenient access to public transport [6]. This convenient access is defined as the walking distance on the road network to public transport stops within 500 m for low-capacity transport systems (e.g., bus) and 1,000 m for high-capacity transport systems (e.g., metro, rail, ferry) [7].

One requirement for this assessment are detailed spatial data concerning the number and location of public transport stops, the road network, and also the place of residence of the local population to analyze the distances which individuals have to travel to access these stops. Data and information about public transport stops are usually sourced from official records maintained by transport authorities (e.g., as General Transit Feed Specification (GTFS) data), or OpenStreetMap (OSM) data [8–10]. Data regarding the length and the layout of the road network is obtainable from OSM. Today, OSM roads are considered to be fairly complete at a global scale, and notably, even better mapped for densely populated places such as urban areas [11], even though some dependence of the data completeness and quality can be still observed with regards to the specific type of dataset or considered region [12,13]. For data-rich countries, population data at a fine spatial granularity are predominantly accessible through national or local census counts, assuming they are made publicly available. However, for many places, especially in developing regions across the world, official population data at a high spatial resolution are barely available. To overcome this limitation, remote sensing data has proven to be an independent source for acquiring precise data on urban settlements which can be used to derive population estimates and can help to mitigate the absence of pertinent population data [14]. At a global scale, remote sensing-based population datasets have been generated in various degrees of spatial granularity, for example for 250x250m grids, i.e., GHS-POP [15] and 100x100m grids, i.e., WorldPop [16,17]. On the contrary to global approaches, also sub-global approaches exist, retrieving population data on continental- [18,19], national- [20,21] or city-scale [22–24]. To measure accessibility, reliable population data are indispensable.

Having outlined the general principles of the accessibility indicator proposed by the UN and the necessary data for its measurement, this introduction will now delve deeper into three key aspects: 1) existing approaches to measure accessibility and evaluations of the UN’s proposed indicator, 2) the specifics of informal and semiformal transport, and 3) the public transport system in Medellín.

### 1.1 Measuring accessibility

The SDG indicator 11.2 proposed by the UN measures the accessibility to public transport in terms of geographical distance. However, accessibility is a comprehensive concept with different manifestations, and for its measurement, a wide range of methods exists. The approaches outlined in the related studies from [2,25] can be categorized as follows: 1) location-based or place-based measures, 2) infrastructure-based or proximity measures, 3) utility-based measures, and 4) person-based measures. The SDG indicator proposed by the UN belongs to ‘infrastructure-based or proximity measures’ and, more precisely, to distance based-approaches using the distance as a direct measurement of accessibility [26]. The indicator presents a practical approach facilitating global comparisons of accessibility to public transport across diverse regions worldwide [6]. It is chosen specifically because of its practicality and broad applicability in diverse regional contexts, especially as it does not depend on highly specialized or context-specific data that may not be readily available in all settings. Unlike other measures that require detailed local data on income, land use, informal transport routes, frequency, or travel times, this distance-based measure facilitates a standardized, accessible approach for comparative studies across global regions. This makes it particularly suited to large-scale or cross-country assessments of accessibility where such localized data is challenging to obtain or inconsistently collected, e.g., the Global South. However, the usefulness and applicability of the SDG indicator is also critically discussed among scholars and practitioners. For example, several studies have addressed the UN’s SDG indicator 11.2 in different geographical and analytical contexts, where the UN’s proposed method was compared with other measures [27,28]. For example, Fried et al. [29] compared the measure proposed by the UN with a location-based accessibility indicator which incorporates income data, travel times, and land use for the case of Nairobi’s minibus system. They emphasize in particular the necessity of including unconventional travel modes such as informal transport options. These are an important mode of transport but due to their informal character they are underrepresented in official data. The authors of [30] investigated the proposed UN measure in two smaller Indian cities, evaluating the resulting accessibility for various formal and informal transportation types across different distances to the nearest transport stop. They recommended the inclusion of informal transport modes and the modification of the accessibility distance of 500 m, particularly for shorter trips. A critical examination of the SDG indicator 11.2 was undertaken by Brussel et al. [31]. The primary critique is that the indicator is more of an approach to measure accessibility to official public transport infrastructure rather than accessibility to activity or opportunity locations. The authors argue that the proposed measure for SDG indicator 11.2 falls short in depicting inequalities in the distribution of accessibility and the complexity of transport systems. Others contend that studies focusing solely on walking distance as an indicator for the quality of public transport might overlook various accessibility barriers such as the urban environment’s quality during walks [32] and factors such as ‘affordability, reliability, gender-based perceptions of safety, crowdedness, unavailability during specific hours, or the inability to reach specific destinations’ [29]. This oversight might result in people who are located within the proximity of the transport stop but choosing not to use it. In particular, the UN’s definition has a narrow scope regarding the types of public transport modes to be included. They even explicitly exclude informal public transport options while precisely including only formal public transport options [5]. However, informal and semiformal transport is a crucial aspect of urban transport for the local population, especially in the Global South [33].

This simplification of urban reality regarding informal transport, semiformal transport and informal settlements, can lead to drawing wrong conclusions from public authorities regarding the state of urban transport. Additionally, the authors of [30] argue for the inclusion of informal and semiformal public transport services or paratransit when considering the local public transport system. This viewpoint aligns with the reality of the existence and usage of informal and semiformal transport modes in many countries worldwide, especially in the Global South [34], where they may even serve as the primary regional public transport option [35].

### 1.2 Informal transport, semiformal transport, paratransit

As previously emphasized, many related studies focus primarily on formal public transport. These are usually operated by official entities, publicly or privately owned, and it adheres to regulations encompassing driver training, compliance with local safety standards, fixed fares, labor protection regulations, predetermined routes and schedules [36]. In contrast, informal and semiformal public transport, also referred to as paratransit, is not operated by official entities [34]. In Latin America, semiformal transport is typically authorized but still operates with a degree of informality, whereas informal transport is often considered illegal, leading authorities to actively combat these services [37]. Informal and semiformal transport services are generally unscheduled and loosely regulated services without designated stops, and routes might be flexible (for the majority of informal services) or largely fixed (for the majority of semiformal services) [36,37]. As a result, these services can adapt swiftly to market and customer demand, offering a level of flexibility that formal services–characterized by high-capacity vehicles and government ownership–often lack [34]. Moreover, informal and semiformal transport options are frequently more cost-effective [38]. The types of informal and semiformal vehicles in Latin America vary considerably and are identified by regional names. These include motorcycles and cars accommodating 3–5 passengers (e.g., ‘Concho’, ‘Taxi colectivo’), minibuses for 10–20 passengers (e.g., ‘Colectivo’, ‘Lotação’), microbuses with capacities of 20–35 passengers (e.g., ‘Buseta’, ‘Chimeco’), and larger buses for 30–70 passengers (e.g., ‘Diablo rojo’, ‘Ejecutivo’) [37]. In other regions of the world the vehicles encompass, e.g., two-wheeler ‘Xe Om’ in Vietnam or ‘Boda-boda’ in Uganda [39,40], three-wheeler ‘Qingqis’ in Pakistan [41], minibuses ‘matatus’ in Kenya or ‘jeepney’ on the Philippines [42,43] or larger buses ‘Molue’ in Nigeria [44]. Informal and semiformal services bridge the gap left by the absence of formal mass public transport and are prevalent in developing countries [34], particularly in urban areas with limited infrastructure, often experiencing rapid population growth and urban sprawl [36,45]. Rapidly growing cities are often characterized by unplanned, low-density settlements at the former urban fringe, the extension of informal settlements, and an increase in travel distances. This results in a decline in the quantity, quality, and condition of public transport services, or their complete absence [36]. In some cases, informal and semiformal transport systems are organized and regulated by local authorities or community-based organizations, allowing them to adopt schedules, routes, and other forms of structure similar to those found in formal transport systems [34]. This form of ‘informal regulation’ can help improve accessibility, particularly in communities that lack the resources to invest in extensive public transportation infrastructure [35]. Often touted as cheaper, faster, and more flexible than formal transport, informal and semiformal transport also demonstrate a higher level of spatial connectivity and plays a significant role in fostering social inclusion for historically marginalized groups [21,37,46]. However, the self- or poorly regulated nature of informal and semiformal transport also invites criticism. Operating vehicles are frequently in suboptimal conditions, raising security concerns. The non-regulatory environment can lead to an oversupply, creating a competitive situation among drivers to be cheaper and faster, often resulting in an exploitative environment for drivers who work long hours for insufficient pay, lacking the means to ensure adequate safety standards for their vehicles [34]. Authorities in many regions, struggle with how to address these concerns—whether to semi-formalize it or combat and suppress it [34]. No general advice can be given how to deal with informal and semiformal transport systems as they exhibit specific and unique characteristics, depending on the regional circumstances [34]. Surveys conducted among residents of informal settlements in Cape Town identified five main barriers to public transport use: 1) walking safety, 2) personal safety, 3) unsafe driving, 4) overcrowding, and 5) walking distance [47]. Therefore, mobility behavior and accessibility to public transport can vary significantly in informal areas.

### 1.3 The situation in Medellín, Colombia

Alongside formal public transport modes, informal and semiformal public transport plays an important role for the urban residents of Medellín, Colombia. The formal public transport system comprises standard buses and the metro network, which consists of metro rail lines, cable cars, and Metroplús buses (a form of Bus Rapid Transit (BRT) system) [48]. In contrast, an essential semiformal option is the minibuses, known locally as ‘Colectivos’. Vague estimations indicate that approximately 250,000 residents rely on these transport services in the metropolitan area of Medellín, which operate across more than 70 sectors in the city [49]. These minibuses have a capacity of 10–20 people [37] and operate as semiformal that meet certain standards but are not fully included into the formal transport system, which is operated by the city of Medellín [50]. These minibuses, accommodating 10–20 passengers [37], operate as semiformal services. While adhering to certain standards, they are not fully integrated into Medellín’s formal public transport system, which is managed by the city [50]. These semiformal services are flexible, with higher coverage and frequency, reducing transfers [37]. Run by smaller private companies, they are authorized to operate specific routes (‘rutas de transporte público colectivo’) under regulatory oversight, complementing the formal system [50,51]. Their routes or destinations are typically indicated on cardboard signs displayed on the vehicle’s windscreen, providing a flexible yet informal service structure [51]. Minibuses usually operate without designated stops and can pick up or drop off passengers anywhere along the route [51]. However, due to the partial integration of the minibuses into Medellín’s official public transport system by local authorities, information on potential minibus stops and their locations is available, indicating a certain degree of regulation and systematization. In addition to semiformal minibuses, informal transport services, such as mototaxis, are also present and are actively targeted by Medellín’s Secretary of Mobility for regulation and control [52]. However, these are not the focus of this study due to the lack of reliable data on this type of Medellín’s informal transport sector. Nevertheless, efforts in other regions aim to address this knowledge gap by developing reliable data on these largely unregulated public transport modes, with the broader goal of integrating them into urban planning frameworks [51,53,54].

Thus, the city of Medellín is suitable as a study area for investigating the accessibility SDG indicator 11.2. Firstly, the public transport system in Medellín consists of formal and semiformal components [55], and secondly, within the metropolitan area of Medellín, there are large areas of informal settlements [56,57]. It renders Medellín an ideal setting, allowing for the thorough examination of the overarching goal of equality embedded in the UN SDGs. The city’s urban layout, with its combination of formal and semiformal transport components and the presence of formal as well as informal settlements, facilitates a focused exploration of the emphasis on the inclusive character of the local transport system within the framework of SDG 11.2. Therefore, we aim at analyzing whether the official accessibility rate based on calculations using the SDG indicator [58] revealing that an overall of 38.35% of Medellín’s population have access to public transport, can be considered a realistic number when semiformal minibuses are included in the analysis.

Compared to formal areas, informal settlements are residential areas characterized by unplanned and unregulated housing often without permission and no security of tenure, as well as a lack of or lower levels of formal basic services and city infrastructure [59]. Informal settlements are inhabited by people from diverse socio-economic backgrounds, ranging from affluent to poor [59]. Mobility behavior in informal areas also differs, as observed in Medellín, where studies revealed distinctive features. Informal areas were found to have a lower number of trips, a lower motorization rate, fewer trips by car, and a higher number of trips by foot and public transport ([55], based on data from origin-destination surveys for Medellín of 2005 and 2011/2012). Furthermore, these marginalized areas lack the same level of spatial connectivity as formal or non-marginalized areas. For instance, Medellín is located within a valley and is surrounded by mountainous landscapes where vast parts of the informal settlements concentrate at these peripheral areas which are characterized by steep terrain [60]. To address this spatial divide, public authorities in Medellín installed cable cars in 2004 with the aim of connecting the peripheral informal settlements situated on the steep slopes to the metro network, thereby promoting spatial connectivity within local communities [60,61]. Further, the authors of [55] conclude that the introduction of the cable car as an integral component of the metro system is advantageous, as it carries the potential to decrease reliance on informal and semiformal transport modes, consequently mitigating security issues associated with these informal services. In the same study, the effects of cable cars on communities in informal settlements are analyzed, unveiling enhanced accessibility through reduced travel time, increased reliability, and even lower costs when compared to alternative bus transportation. However, they noted that affordability constraints persist, potentially limiting the attractiveness of cable car usage for residents of informal settlements in Medellín. Notably, the responsible authorities have continued to enhance the metro system by introducing new cable car lines, with developments extending until the year 2021 [48]. In Medellín, there are periodic controls to reduce unlicensed informal vehicles [49]. In fact, many people continue to use informal and semiformal transport, as formal transport modes often cannot meet the demand and are unequally distributed across city space. Examples from Colombia illustrate that despite the extensive expansion of official public transport, the reliance on informal and semiformal transport persists [62].

Against these backgrounds, this paper endeavors to address the following research inquiries focusing on the specific case of Medellín:

1) How does accessibility geographically vary throughout the city?
2) Does accessibility exhibit variations across different formal and semiformal transport modes? To what extent does informal public transport contribute to overall accessibility to public transport?
3) What role has the expansion of the metro system in Medellín (2016 vs. 2021) played in enhancing accessibility to public transport?
4) Does the accessibility to public transport differ in informal settlements compared to formal settlements, and how is accessibility correlated with socio-economic factors?
5) Can remote sensing data and related techniques serve as a viable solution to bridge the data gap in areas lacking population data, particularly in regions with data scarcity, such as the global south? This will be compared to disaggregated population in cadastre buildings.

To address these questions, we calculate the SDG indicator 11.2 proposed by the UN for the city of Medellín in terms of measuring accessibility by using the walking distance to the next transport stop. Further, it is analyzed how accessibility to public transport can spatially differ throughout the urban area by comparing the median walking distances at three different spatial scales: 1) on the entire city level (comparable to the approach proposed by indicator 11.2), 2) within administrative units throughout the city (sectors), and, 3) within raster grid cells. Moreover, we investigate how remote sensing data can help in assessing these accessibilities by incorporating three different types of population data: 1) Population data based on disaggregated census data to cadastre buildings [14], 2) a fine-granular remote sensing-based population data set at the level of single buildings [24], and, 3) WorldPop data, a globally available 100x100m remote sensing based population product [17]. To the best of our knowledge, this has not been previously attempted, offering a potential improvement and simplification of methodologies for future accessibility assessments. As Medellín offers formal and informal and semiformal transport types, and as these modes have not been studied so far in the glimpse of accessibility, we separately consider the accessibility to the different types of transport systems. Furthermore, in the context of the social implications of public transport [1], we analyze the differences of accessibility in regard to socio-economics and formal vs. informal settlement types.

In Section 2, we present an overview of the study area of Medellín, describe the used data and methodology for our analysis. The results are presented in Section 3, which are discussed in detail in Section 4. The conclusions of our study are given in Section 5.

## 2. Data and methods

### 2.1 Study area and region of interests

Medellín is the second most populated metropolitan area within Colombia with approximately 4.2 million inhabitants. The responsible authorities took early action, initiating public transport development and inaugurating the first metro line back in 1991. Since then, the public transport system has seen significant expansions, incorporating not only additional bus routes but also introducing more metro lines, along with several cable car lines [48]. The inaugural cable-car line in Medellín was constructed in 2004, connecting informal areas in the northeast to the pre-existing north-south overground metro line [63]. Such complex public transport infrastructures can rarely be found among cities in Latin America. In contrast, the Colombian capital Bogotá with over 10 million inhabitants in its metropolitan area relies solely on a street-based rapid bus system [64]. Due to the early establishment of the public transport system in Medellín and its constant extension, Medellín offers an ideal basis to serve as a study area.

**Fig 1:**
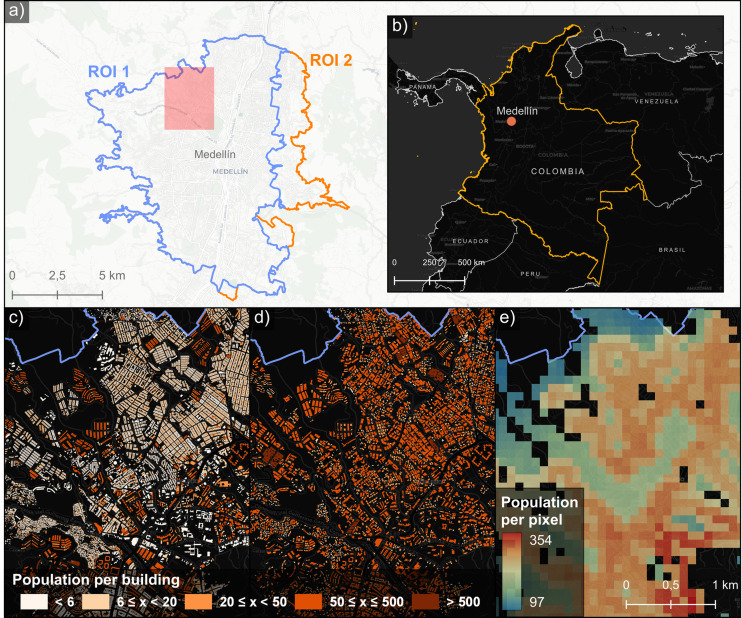
Overview of study area and used population datasets. (a) ROIs in Medellín: ROI 1 (blue) and ROI 2 (orange); (b) location of Medellín; (c) population dataset 1: census-based population data per cadastre buildings; (d) population dataset 2: remote sensing-based population per building; and, (e) population dataset 3: WorldPop raster data (100x100 m). *Basemap © CartoDB, licensed under CC BY 3.0. Data © OpenStreetMap contributors, available under ODbL*.

The considered region of interest 1 (ROI 1) covers a large part of the Municipality of Medellín with an area of approximately 96 km² (Fig 1a). It consists of a total of 200 administrative units, called ‘sectors’ (*sectores* in Spanish), with almost 1.8 million inhabitants according to official census data for 2018. The ROI 1 was chosen based on the joint coverage of the three population datasets used and compared in this study. The terrain within this study area is very heterogenous, as the city of Medellín is located in the Aburrá Valley surrounded by mountains on both sides (Fig 1b). An extended ROI (ROI 2) was used for the analyses incorporating socio-economic data and the location of informal settlements covering the whole city of Medellín (further described in Section 2.4 and 2.5).

### 2.2 Population data

In this study, we compare three different types of population datasets to evaluate the effect on the degree of accessibility. These datasets represent various levels of detail of spatial granularity (Fig 1 b,c,d; Table 1). This comparison allows us to assess two critical aspects: first, whether remote sensing-based datasets can provide a valuable alternative to official census data in data-scarce regions, and second, the extent to which global datasets like WorldPop can approximate accessibility assessments in diverse urban settings despite their inherent limitations.

**Table 1:** Overview of the used population datasets. All three population datasets are available for ROI 1, whereas dataset 2 is not available for the extended study area ROI 2.

	dataset 1: population data from cadaster [14]	dataset 2: fine-scaled remote sensing based population data [24]	dataset 3: global-scaled remote sensing based population data [17,65]
**Population count within ROI**	1,770,534	1,762,586	2,441,854
**Description**	population data on building level created by using cadaster buildings and census data	population data on building level created by using remote sensing and open data	global population data with 100x100m grid cell size

**First**, we use vector-based population data at building level based on census population, land use and cadaster buildings from [14]. The population per building was obtained by using a top-down population disaggregation approach by using official census data, by incorporating the number of floors per building and official land use information. This dataset can be considered as a highly accurate population dataset (dataset 1). **Second**, we use a fine-scale, vector-based population dataset based on a remote sensing and open data approach [24]. It combines a deep learning building extraction approach from aerial remote sensing imagery, height information from satellite data, and number of floors estimated with a regression model using Google Street View (GSV) data and land use data from OSM to retrieve the number of people per housing unit (dataset 2). **Third**, the globally available WorldPop population dataset is used [17], specifically from 2020 for Colombia [65], represented as a raster with a cell size of approximately 100x100 m (dataset 3).

### 2.3 Data on public transport stops

**Fig 2:**
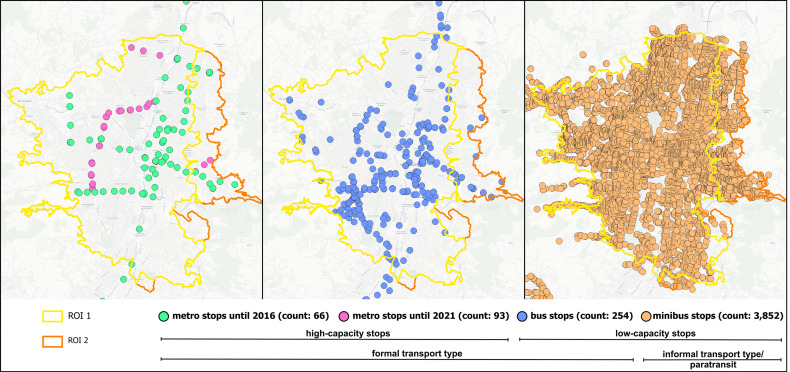
Locations and types of transport stops, count of stops per type provided in brackets.

We utilized public transport stop data from various sources, incorporating both low-capacity and high-capacity transport stop data (Fig 2). As stated in the introduction, GTFS data are one of the main sources for acquiring public transport stop locations. However, only 2.45% of the Latin American travel agencies publish their public transport information as GTFS feeds [9]. To overcome this limitation, we employed GTFS data from [66] to capture the locations of formal public transport, refining them by eliminating redundant and irrelevant entries. Additionally, we updated this data with information from the local transport agency ‘Metro de Medellín’, incorporating 31 new metro stops introduced after the last available GTFS data in 2016. This update accommodated the significant expansion of the metro network with the addition of new lines M (Feb 2019), O (Nov 2019), and P (June 2021). Thus, for a comprehensive analysis of the impact of these newly installed metro lines, we utilized two sets of metro location data: metro stations for 2016 and metro stations for 2021. Minibus stop data, constituting a substantial portion of the public transport stops, were obtained from local authorities in Medellín. The location and the count of the transport stop data per transport type within the study area can be seen in Fig 2.

### 2.4 Socio-economic data

**Fig 3:**
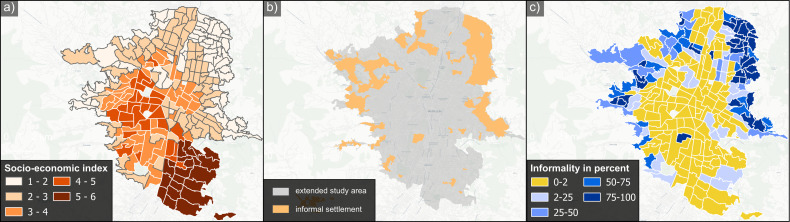
Extended study area ROI 2 for socio-economic analyses. (a) socio-economic data by sector; (b) location of informal settlements; (c) percentage of informal settlements per sector. The socio-economic index ranges from 1 (‘very low/poor’) to 6 (‘very high/rich’), and the informality percentage represents the proportion of sector area covered by informal settlements. *Basemap © CartoDB, licensed under CC BY 3.0. Data © OpenStreetMap contributors, available under ODbL*.

To examine the influence of socio-economic factors on public transport accessibility, we leveraged socio-economic data available for the extended ROI 2, as depicted in Fig 3. The socio-economic data was sourced from the official census conducted by the government of Medellín in 2018 [67]. These include information on the number of households within each sector, categorized into one of the six socio-economic groups (*estrato socioecónomico*). These categories span from 1 (‘very low’ or ‘very poor’) to 6 (‘very high’ or ‘very rich’).

To derive a socio-economic index for each sector, we calculated the mean value using the number of households belonging to specific socio-economic groups (Fig 3a). This resulted in the classification of the sectors into five socio-economic groups, each associated with a distinct socio-economic index: group 1 (‘very low’ with an index <2), group 2 (‘low’ with an index 2–3), group 3 (‘medium’ with an index 3–4), group 4 (‘high’ with an index 4–5), and group 5 (‘very high’ with an index 5–6).

### 2.5 Informal settlements data

The locations of informal settlements within the extended ROI 2 of Medellín are depicted in Fig 3b. These informal settlements often occupy steep slopes, exposing them to a higher risk of landslides [57]. The data for informal settlements are sourced from the Land Use and Zoning Plan 2014, Plan de Ordenamiento Territorial (POT), published by the city of Medellín [68].

Within this dataset, two categories, namely Comprehensive Improvement (Mejoramiento Integral, MI) and Consolidation Level 3 (Consolidación Nivel 3, CN3), were selected as informal settlements based on the city administration’s classification of them as precarious settlements [69], a designation also used by Kühnl et al. [56]. We classify the sectors into five classes based on the percentage of informal settlements they contain, and additionally, into formal or informal, where informal sectors have at least 2 percent of informal settlements. We use these categories to explore the relationship between the percentage of informality within sectors and the walking distance to the nearest transport stop, and to compare the distances in the formal and informal sectors (Fig 3c):

- <2% coverage of informal settlements: considered as formal
- 2–25% coverage of informal settlements: barely informal
- 25–50% coverage of informal settlements: moderately informal
- 50–75% coverage of informal settlements: mostly informal
- >75% coverage of informal settlements: highly informal

### 2.6 Scenarios for the comparative analysis of accessibility

We use an open source Python package for routing and calculating the distance to the closest public transport stop and the accessibility according to the SDG 11.2 indicator [70]. The three population datasets were converted to point data using the centroid of the corresponding building or cell, to be accordingly prepared to be used within the package. These population points are the starting points for routing the walking distance via the OSM street network to the public transport stops. This results in a calculated distance to the next public transport stop per population point.

For analyzing how the accessibility to the public transport stops differ throughout the city, we define the following analysis strategy: initially we aim at comparing formal and semiformal transport stops and their impact on the accessibility. Therefore, first, we analyze only formal public transport stops including metro and bus stops (Section 3.1), and second, we extend the analysis to include also semiformal minibus transport stops (Section 3.2). This analysis is followed by an attempt to gain a better understanding of the role of low (bus and minibus) and high capacity transport modes (metro) (Section 3.3). Finally, we delve into the analysis of various socio-economic groups, taking into account formal and informal settlements and their accessibility to urban transport (Section 3.4).

For the different analyses in the experimental setup, we compare the calculated walking distance to the public transport stops based on three population datasets (cp. Section 2.2) at three different spatial levels: 1) the overall accessibility for the entire city area of ROI 1, which depicts the measure according to the SDG 11.2 indicator, 2) the accessibility within administrative units throughout the city (sectors), and, 3) the accessibility within a regular grid with a cell size of 1,000 m.

## 3. Results

### 3.1 Accessibility based on formal public transport

The formal public transport system in Medellín comprises the metro and bus stops, which serve to measure the accessibility according to SDG target 11.2, with a walking distance of 1,000 m and 500 m, respectively (Table 2a). Analyzing the accessibility outcomes exclusively based on population dataset 1 (Table 2.1), we observe that the bus system exhibits the lowest accessibility, yielding a share of 32.4% of the population. This translates to approximately 570,000 individuals residing within a 500 m walking distance of bus stops. In contrast, accessibility to metro stations is reported at 36.7%, with a notable increase to 48.6% following the implementation of new metro lines until the year 2021. Consequently, an additional 11.9% of the total population now has access to high-capacity transportation options compared to the baseline in 2016, which equals an estimated increase of nearly 210,000 individuals having gained access to public transport. This effect of increased accessibility is also observed for the combined accessibility of metro and bus stations for the year 2021, where this positive trend is also evident resulting in an overall increase of accessibility of 9.3%, which can be translated to a total of 1.1 million out of 1.8 million inhabitants (based on dataset 1) of the considered ROI 1 benefiting from formal public transport in Medellín. This highlights a substantial improvement in public transport accessibility since the inception of the new metro lines, which play a pivotal role in increasing overall accessibility within the formal transport system. However, it is crucial to acknowledge the complementary role of bus stops, as of 2021, they are contributing significantly to the combined accessibility of metro and bus stations, which stands at 62.8%. This surpasses the 48.6% accessibility provided by metro stations alone.

**Table 2:** SDG-Accessibility in percentage and total amount of individuals with access to public transport according to each transport type in ROI 1 for (a) formal and (b) semiformal transport. The analysis is conducted using population data from three distinct datasets: 1) cadaster, 2) remote sensing: fine-scaled regional approach, and 3) remote sensing: global approach.

					Population datasets			
			1) dataset 1		2) dataset 2		3) dataset 3	
Total population count within study area			1,770,534		1,762,586		2,441,854	
			*in %*	*in total*	*in %*	*in total*	*in %*	*in total*
	**Type of stops**							
a)	Formal	metro (until 2016)	36.7	650,494	40.2	708,207	39.8	972,835
		metro (until 2021)	48.6	860,480	52.1	919,012	51.7	1,262,683
		Bus	32.4	574,124	40.3	709,850	41.6	1,015,789
		metro & bus (until 2016)	53.5	947,590	58.8	1,036,224	60.5	1,476,101
		metro & bus (until 2021)	62.8	1,112,427	67.6	1,191,332	68.9	1,681,217
b)	Semiformal	Minibus	98.9	1,750,881	98.2	1,731,564	96.8	2,363,715

Comparing the results using different population datasets, dataset 1 serves as a baseline for comparison with datasets 2 and 3. When examining the relative results of these datasets, only slight deviations are observed depending on the type of transport considered. When comparing to population dataset 1, however, the total number of persons considered to have access to public transport is particularly overestimated when using the global population dataset 3. In contrast to dataset 3, population dataset 2 is more accurate in estimating the total number of persons with access to public transport.

**Fig 4:**
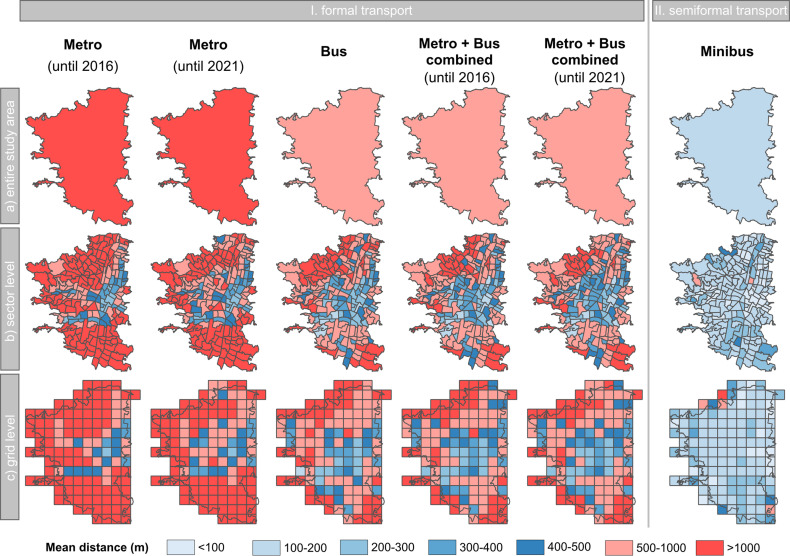
Mean distance to the closest public transport stop in ROI 1. Mean distances (in meters) to I) formal and II) semiformal transport by transport type at (a) the entire study area level, (b) the administrative level, and (c) the grid level, using population dataset 1.

In general, the calculation of the indicator for SDG target 11.2 is performed at the level of the entire city area. However, this approach tends to obscure the nuanced geographical and spatial variations in accessibility within the heterogeneous urban context. In order to delve into these intra-urban differences, we conduct a comparative analysis. Specifically, we assess the mean walking distances to the nearest public transport stops across the whole area of ROI 1 using population dataset 1 (Fig 4a). To further elucidate these distinctions, we compare the distances at both the administrative sector level (Fig 4b) and on a grid-level with a cell size of 1,000 m (Fig 4c).

Examining the formal transport types at the entire ROI 1 level (Fig 4.I.a), the effects of newly implemented metro lines post-2016 is notably recognizable in the mean walking distance to the nearest metro station. The introduction of these lines led to a significant reduction in distance, decreasing from the initial 1,571 m in 2016–1,283 m in 2021 which means that on average residents have to walk 288 m less to reach a metro stop. Nevertheless, by nature the mean walking distance to the nearest bus station is shorter, measuring only 801 m. Overall, the combination of both metro and bus stops yields the shortest mean distance, registering 741 m in 2016 and further reducing to only 699 m in 2021. This highlights that the newly established metro stops after 2016 contribute to a notable decrease in the mean walking distance to any formal transport stop, being it metro or bus, by an additional 42 m resulting in a higher share of the population being able to have access to public transport.

In contrast to the analysis at the entire ROI 1 level, the more granular spatial analyses conducted at the sector and grid level (Fig 4.I.b and 4.I.c) unveil significant variations in the mean walking distances to the nearest formal transport stops across the study area of Medellín. A discernible spatial pattern emerges, with central areas exhibiting shorter distances to the nearest transport stop, while greater distances characterize the outer regions of the study area. A closer qualitative examination of metro stops for both 2016 and 2021, at both sector and grid levels, reveals substantial improvements in accessibility, particularly in the northern and central-western parts of Medellín. These areas benefit most from the extension of the metro system after 2016. This effect is also present for the combination of the metro and bus stops, resulting in a further reduction of distances. Furthermore, the bus system is able to close some accessibility gaps between the metro stations, effectively reducing walking distances in between the metro stations, especially in and around the central core of the study area.

### 3.2 Accessibility based on informal public transport

An integral component of Medellín’s public transport system is the semiformal transportation network, specifically represented by minibuses. The extensive network of minibus stops forms a dense coverage of transportation options throughout the study area, with a total of 3,852 stations (according to the used minibus stop data from the local authorities in Medellín). In order to assess their effect on public transport accessibility, analogous analyses to those conducted for formal public transport were undertaken.

The accessibility of minibus stops, calculated as a percentage based on population dataset 1, stands at 98.9% (Table 2b). This translates to accessibility for more than 1.75 million individuals in the study area, all reachable within a 500-meter walking distance – an approximation of the study area’s entire population on the basis of population dataset 1 (cp. Table 1). Additionally, the mean walking distance to the nearest informal stop across various spatial entities was computed (Fig 4.II). With a mean distance of 156 m the minibus stops can be reached at the level of the entire study area (Fig 4.II.a). This pattern, based on a qualitative examination of Fig 4.II.b and 4.II.c, persists across the investigated administrative and grid entities, where the mean walking distance is notably lower compared to formal transport types. At the sector level, the central-eastern part of the study area stands out with the shortest mean walking distance – less than 100 m to the nearest public transport stop, underscoring the high accessibility of minibuses in this region (Fig 4.II.b).

### 3.3 Accessibility based on socio-economic level and informality

Based on population dataset 1 and the extended ROI 2, we further analyzed the effect of socio-economic groups and the type of settlement (formal vs. informal) on the median of the mean walking distance. This was done per sector to the next public transport stop per transport type represented as boxplots. Therefore, in the following, the median of the mean walking distances per sector is given.

**Fig 5:**
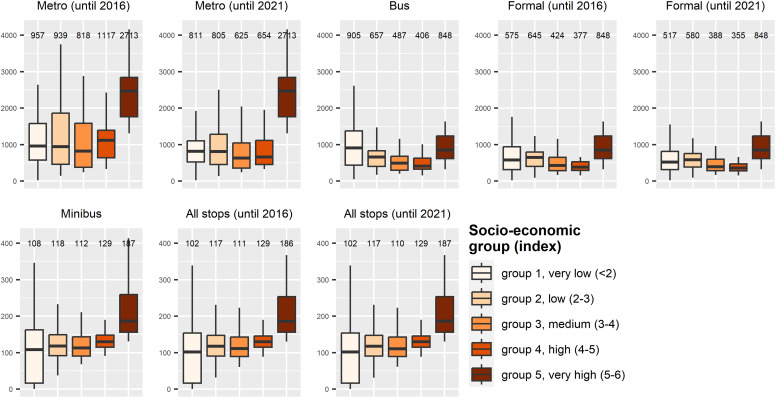
Walking distances to the nearest transport stop by socio-economic level in ROI 2. Comparison of walking distances (y-axis) from dwelling units to the closest transport stop by mean socio-economic level (x-axis) per sector, ranging from very low (<2) to very high (5–6). Median distance values are given above each boxplot. Note the altered y-axis limits in upper (4200 m) and lower plot row (420 m).

A comparison of the socio-economic index within the sectors and the closest distance to the next transport stops from each dwelling unit is presented in Fig 5. The derived median walking distances per socio-economic group (shown above each boxplot) show that distances are larger for all transport types for the very high socio-economic groups between 5 and 6 (very high), except for the transport type ‘bus’ (median distance of 848 m). For the transport type ‘bus’, the sectors with the lowest socio-economic group exhibits the largest distances (median distance of 905 m). The newly installed metro stations until 2021 reveal an improvement in accessibility in general. For all socio-economic classes, except for class 5–6 (which remains the same), shorter median distances are measured. Considering the formal transport types, the official bus system is a suitable add-on to the metro system, as it is able to further decrease the median distance to the closest transport stops. The median distance of all transport stops (formal and informal) changes only slightly (cf. Fig 5, lower boxplot series) over time. This emphasizes the importance of the minibus system for the accessibility to public transport in Medellín. The new metro stops shortened the median distance by 243 m compared to 2016 for all socio-economic groups.

Table 3a provides an in-depth analysis of the impact of the newly installed metro stations until 2021 on the median walking distance across the investigated socio-economic groups. Group 4, with a high socio-economic index, experienced the most significant benefits from the expansion of the metro system between 2016 and 2021, witnessing a substantial 41.5% decrease in walking distance to the nearest metro station. Following this, the other socio-economic groups exhibited varying degrees of improvement: group 3 demonstrated a 23.6% decrease, group 1 experienced a 15.3% reduction, group 2 observed a 14.3% decrease, and group 5 showed no change in the median distance to the next metro stop (explanation: there was no extension of the metro system in sectors of socio-economic group 5 between 2016 and 2021). Different patterns emerge when considering the results for all formal public transport stops, encompassing both metro and bus stations. Among the lowest socio-economic groups, group 1 and group 2 experienced the most significant advantages, with a reduction in walking distance of 10.1%, followed by group 3 with an 8.5% decrease, and group 4 with a 5.8% improvement.

**Table 3:** Overview of differences in median walking distance in percent per category/group to next public transport stop resulting from the extension of the metro network after 2016. Negative values indicate a decrease of median walking distance per sector per category. Categories: (a) mean socio-economic index per sector, (b) type of settlement per sector, and, (c) percentage of informality per sector.

category/ group	Difference of median distance 2016 vs. 2021 [%]	
	Metro	formal
**a) Mean socio-economic index per sector**		
Group 1 (<2)	-15.3	**-10.1**
Group 2 (2–3)	-14.3	**-10.1**
Group 3 (3–4)	-23.6	-8.5
Group 4 (4–5)	**-41.5**	-5.8
Group 5 (5–6)	0.0	0.0
**b) Type of settlement per sector**		
Formal	**-28.8**	**-11.5**
Informal	-6.1	-6.3
**c) Percentage of informality per sector**		
<2%, not informal	-28.8	**-11.5**
2–25%	-14.4	-7.5
25–50%	**-31.6**	-10.5
50–75%	-7.0	-2.0
75–100%	-7.4	0.0

Note: The highest number per metro or formal stops are formatted in bold.

It was also investigated if the type of settlement influences the mean walking distance to the nearest public transport stop (Table 4). An important finding is that for the formal public transport stops combined (metro and bus), the median distance to the nearest stop is larger for those living in sectors with informal settlements. On the contrary, for semiformal transport stops and when combining formal and semiformal stops, distances are slightly shorter for informal settlement types, although these differences are small. The new metro lines decreased the distances for both, formal and informal settlement types. However, formal settlement types profited much more from the additional metro lines, from which the median distance decreased from 940 m in 2016–698 m in 2021, which depicts a reduced distance of 242 m. On the contrary, in informal settlements, only a reduction of 61 m can be observed. Table 3b also shows that residents of formal settlements experience a stronger decrease in median walking distance (28.8% to the nearest metro station and 11.5% to the nearest formal public transport station), in contrast to individuals residing in informal settlements (6.1% and 6.3%, respectively).

**Table 4:** Comparison of median distances to the considered public transport stops in regard to formal or informal sector/settlement type.

	Formal sector	Informal sector
**Type of transport**	*median distance to next public transport stop [m]*	
Metro (until 2016)	980	997
Metro (until 2021)	698	936
Bus	514	752
Formal transport (bus and metro until 2016)	479	650
Formal transport (bus and metro until 2021)	424	609
Minibus (semiformal transport)	128	115
All stops (formal and semiformal until 2016)	127	114
All stops (formal and semiformal until 2021)	127	114

**Fig 6:**
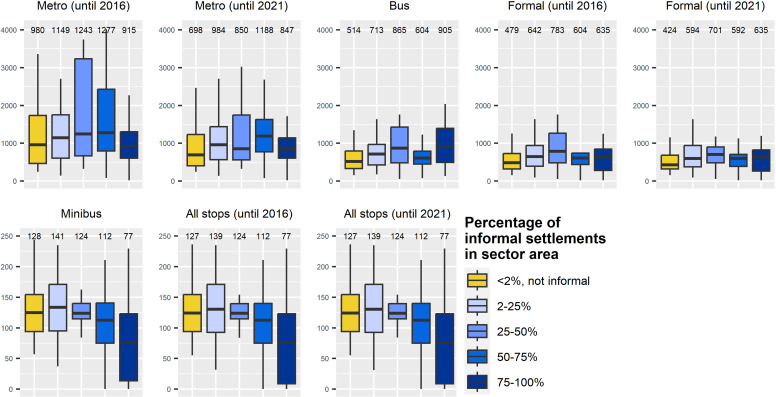
Distance to the closest public transport stop by percentage of informal settlements. Boxplots showing distances to the closest public transport stops in meters (y-axis) by percentage of informal settlements per sector, grouped into five classes. Median distance values are given above each plot. Sectors with less than 2% informal settlements are considered formal. Note the altered y-axis limits in upper (4,050 m) and lower row (250 m).

To provide a more detailed picture, the degree of informality within a sector was determined presenting five different groups of sectors, ranging from formal to informal based on the percentage of informal settlements (Fig 6). The additional metro stations in 2021 reduced the median distance compared to 2016. We found, from more to less, a reduction of 393 m for sectors with 25–50% informality, 282 m for the formal sectors, 165 m for sectors with 2–25% informality, 75 m for sectors with 50–75% informality, and 68 m for the sectors with 75–100% informality. Therefore, the sectors with a higher percentage of informal settlements benefit the least from the metro extension. The official bus system and the formal transport types in general are more accessible to people living in formal sectors. On the contrary, the semiformal minibus system is more accessible (shortest distance to minibus stops) by people living in sectors with a share of 75–100% informal settlements, underlining the importance of this semiformal transport mode for these sectors.

## 4. Discussion

In this study, our focus was on examining the accessibility of public transport in Medellín, specifically in relation to the walking distance to the nearest public transport stop, as defined by the UN. Here, we aim to provide a concise summary of the main findings in line with the established research questions, supported by key evidence. Additionally, we will draw connections between our findings and the existing literature, delving into the strengths and limitations of our study. Ultimately, we will conclude by encapsulating and emphasizing the fundamental aspects of our research.

For **research question 1**, we found a substantial geographic disparity in accessibility across Medellín. Our findings indicated that the central urban core consistently demonstrates the highest accessibility rates for all examined modes. Conversely, regions situated at the periphery of the urban area exhibit lower accessibility levels, highlighting the pronounced geographical inequality in accessibility distribution throughout Medellín.

Concerning **research question 2**, our investigation revealed a substantial variation in accessibility among the distinct formal and semiformal transport modes. Utilizing the population dataset derived from the census (dataset 1), we observed that the accessibility to public transport is most pronounced for the semiformal minibus mode, reaching 98.9%. Notably, this figure surpasses the combined accessibility of all formal transport stops, encompassing both bus and metro stops, which stood at 62.8% in 2021, and the calculated accessibility of 38.35% by UN-Habitat [58]. These findings underscore the considerable significance of semiformal transport modes in shaping the accessibility landscape of public transport in Medellín.

Another key finding in regard to **research question 3** was the substantial enhancement in accessibility resulting from the expansion of the metro system between 2016 and 2021. The accessibility to metro stations alone witnessed a remarkable increase, with 11.9% more residents deemed access to this high-capacity transport mode. This positive effect was also evident in the overall accessibility to formal transport stops, encompassing both metro and bus stops, where an additional 9.5% of the population could be considered to have access to public transport.

Moreover, concerning **research question 4**, accessibility to public transport is closely linked to socio-economic factors. Notably, the high socio-economic group (group 4) experienced the greatest benefits from the metro extension post-2016, with a 41.5% reduction in walking distance. In contrast, the lowest socio-economic group (group 1) saw the smallest improvement with 15.3% reduction. This underscores that while local authorities succeeded in reducing walking distances across socio-economic groups, the benefits of accessibility are not evenly distributed. Additionally, accessibility varies according to the type of settlement. Across all public transport modes, residents of informal settlements face poorer accessibility. For instance, the walking distance to formal public transport modes is 185 m longer for residents of informal settlements (609 m) than for residents of formal settlements (424 m) (based on formal stops for the year 2021). Furthermore, it was also found that communities living in sectors with the highest proportion of informality (50–75% and 75–100%) benefited the least from the metro system extension. However, the group that benefited the most were those living in sectors with 25–50% informality. Thus, once again, the benefits are not evenly distributed among all residents, underscoring the crucial role of the minibus system for communities in informal areas which was best reached by residents living in sectors with a proportion of 75–100% informality (distance of 77 m) followed by sectors with 50–75% (distance of 112 m) and 25–50% (distance of 124 m) of informality.

We found that the semiformal minibus system in Medellín is crucial for connecting the local communities from informal settlements to the city’s public transport system. The formal public transport system would not allow the informal dwellers easy access to the city center. Due to the increase in accessibility to public transport by the semiformal system, the connectivity of these communities to essential city services, such as jobs and education is enhanced [34]. Furthermore, authorities in Medellín have increased accessibility within the city’s informal areas by extending the metro line, particularly through the introduction of new cable car lines. The effectiveness of the cable car in Medellín in connecting people to the city has already been demonstrated [55]. However, our findings revealed that communities with low socio-economic levels still have to walk longer distances to reach these efficient, high-capacity formal modes. Thus, inequality in accessing safe, reliable and regulated public transport persists and has not been mitigated by the metro system extension. Providing safe public transport to all, regardless of income and residence location, is essential to prevent social exclusion, particularly in the Global South [31]. Therefore, it is crucial to promote transport planning that aims for equal accessibility rates for marginalized groups, especially to enhance the transformative effect on the community [55].

For **research question 5** our study revealed that population data generated using remote sensing data and techniques serve as a feasible alternative in the absence of official population data. Particularly, approaches incorporating regional circumstances (dataset 2) can be preferred over global population datasets (dataset 3), as they demonstrate a higher overall alignment with the reference population dataset 1. This finding highlights the potential of alternative population datasets to enhance accessibility analyses, particularly in data-scarce environments. By leveraging unconventional data sources such as remote sensing, our study provides a more nuanced understanding of population distribution, which is critical for addressing urban accessibility challenges in regions like the global south.

To the best of our knowledge, this is the first study to integrate remote sensing-based population data into public transport accessibility analyses for comparing population data of varying granularity for this purpose, and benchmarking these remote-sensing data against official population datasets for this specific domain. This represents a significant contribution to the existing literature, as it underscores the applicability of remote sensing techniques to bridge critical data gaps in urban accessibility studies, particularly in regions where traditional census data are unavailable or unreliable. Moreover, our findings align with prior observations by the authors of [71], who emphasized that two cities with similar overall population densities can have vastly different transportation demands due to variations in population distribution. Our study provides empirical evidence supporting this claim by demonstrating how regional-specific population datasets (dataset 2) better capture the spatial distribution of populations compared to global datasets (dataset 3), which often overlook local variations. This nuanced approach could lead to more effective and equitable transport planning and accessibility assessments in rapidly urbanizing areas.

Our study provides critical empirical evidence that underscores the importance of the semiformal public transport system in contributing to overall accessibility to public transport in Medellín. By highlighting the significant role of these systems, which often fill gaps left by formal public transport networks, our findings address a key aspect of public transport accessibility in developing cities [34,72,73]. Additionally, our research adds valuable insight into the pronounced disparities in accessibility to public transport between formal and informal settlements [2,74], providing a more nuanced understanding of the spatial and socio-economic inequalities in urban transport provision. Moreover, we provide empirical evidence on the clear improvements in accessibility to formal public transport modes, specifically the expansion of the metro system through the addition of new cable car lines, achieved through strategic expansions by public authorities over a five-year period. This improvement is especially impactful for residents of informal settlements, demonstrating the role of targeted infrastructure development in reducing accessibility disparities in Medellín.

Together, these contributions not only advance the understanding of urban transport systems in data-scarce regions but also offer actionable insights for policymakers aiming to foster equitable and inclusive urban mobility. The revealed accessibility patterns identify regions that remain underserved by formal public transport, offering initial guidance at the spatial levels of sectors and grid cells to inform decision-making on where to prioritize accessibility improvements. This is particularly evident in the urban fringe areas (cp. Fig 4b and 4c]. While the extension of the metro system has increased accessibility to formal public transport (cp. Table 4), the benefits have been disproportionately greater for residents with higher socio-economic status living in formal settlements compared to those with lower socio-economic status residing in informal settlements (cp. Table 3). This underscores the urgent need for local authorities to take targeted action to reduce disparities in public transport accessibility, with a particular focus on improving mobility options for marginalized groups.

In this study, we calculated accessibility to public transport for various scenarios, considering the influence of available population data, transport modes, socio-economic factors, and informality. However, the robustness of our analyses depends entirely on the quality and reliability of the data used. The impact of data quality on our results cannot be neglected, as we rely, for example, on open source transport stop data – with unknown completeness or accuracy. A major drawback of open source data is their highly variable quality [75], yet such data frequently serve as the only alternative when official data are unavailable. Similarly, the used population datasets from each source exhibit considerable variability, which could influence the robustness of our conclusions. By incorporating data from various sources and levels of detail, such as different population datasets, our analysis highlights the extent to which these variations affect the results of the accessibility assessment.

Additionally, the calculated accessibilities cannot be directly compared to other studies or reference data. The only comparison can be made with the UN-Habitat’s calculation, which found that the accessibility of formal transport is 38.35%, compared to our 62.8%. Differences may be attributed to the use of different population data, street network data, public transport stop data, study area extents, or methods applied. Our geographically detailed analyses revealed significant variability in accessibility throughout Medellín, which the UN’s measure, considering the city as a uniform area, does not capture.

While this study focuses on the accessibility of public transport in terms of walking distance to stops, we acknowledge that temporal factors such as frequency, timing, and reliability play a crucial role in shaping transport accessibility. However, these data are often unavailable in contexts characterized by semiformal and informal transport systems [54], as is the case in Medellín. Despite these limitations, it is essential to recognize the importance of reliability and service quality for public transport accessibility [34]. Authorities must prioritize collecting and integrating these data to enhance transport accessibility and equity in underserved areas.

An important limitation of this study is the use of cross-sectional data for both population and socio-economic variables from the years 2016 and 2021. While this approach allows for assessing the impact of the metro expansion on accessibility between these two points in time, it assumes that population and socio-economic characteristics remained constant during the period of metro system expansion. However, socio-economic conditions and population dynamics may have changed during this time frame, which could influence the results. Future studies could benefit from using longitudinal data, if available, to capture changes in socio-economic and population characteristics alongside the expansion of public transportation infrastructure.

While environmental factors, such as weather conditions and temperature variations, can influence public transport accessibility–particularly in regions with significant seasonal or climatic variability–these factors were not included in our analysis. This study focused on Medellín, a city with relatively stable temperatures due to its equatorial location, but subject to bimodal rainy seasons in April–May and October–November. Periods of heavy rainfall may affect accessibility, especially in areas prone to flooding or with less resilient infrastructure. By focusing on structural and socioeconomic influences, we did not account for these environmental variables. Future studies examining multiple cities or regions could incorporate such factors to provide a more comprehensive understanding of their impact on public transport accessibility.

In this study, we used a routing method instead of the Euclidean distance. However, accessibility barriers or individual walkable neighbourhoods [76,77] are not considered in the distance-based approach used by both, the UN and our study. Accessibility barriers, such as the quality of the urban environment on the way to public transport stops, include affordability, reliability, gender-based perceptions of safety, crowdedness, unavailability during specific hours, or the inability to reach specific destinations [32]. We also explored the use of remote sensing as a potential source for population data. Projected remote sensing-based population data are available at national or global levels [78], making it highly promising for future modelling of accessibility to public transport (e.g., the authors of [29] used WorldPop data for accessibility analyses).

## 5. Conclusions

Our primary goal in this study was to investigate accessibility to public transport by examining the effects of various transport modes (high-capacity vs. low-capacity, and formal vs. semiformal transport modes), different scales (entire city vs. administrative areas vs. grid areas), socio-economic factors, and settlement types (formal vs. informal settlements). We found that all these factors significantly impact the level of accessibility to public transport, and that city-wide approaches often overlook these local nuances. Informal and semiformal transportation systems are crucial in the Global South, especially for poor communities. These systems highlight the creativity and business acumen of residents who compensate for the lack of formal transport services. This situation reveals two key points: first, the areas of the city that are underserved by formal transport are largely informal; second, it may indicate that city administrations tend to favor wealthier social classes.

Future research should focus on integrating more granular data and exploring innovative solutions to further enhance accessibility to (formal) public transport. By doing so, urban planners and policymakers can better address the socio-spatial inequalities that affect the most vulnerable populations, contributing to more inclusive and sustainable urban environments.
